# Diseases, Etc. of the Rectum and Anus

**Published:** 1860-11

**Authors:** 


Art. VI.—*On the Diseases, Injuries, and Malformations of the Rectum and Anus, with Remarks on Habitual Constipation.* By T. J. Ashton, Surgeon to the Blenheim Dispensary, etc. etc. Third edition. 8vo., pp. 420. London, 1860.

The demand for a third edition of this work, so soon after the appearance of the second, while it shows a thorough appreciation of its merits by the profession, must be highly gratifying to Mr. Ashton, who has evidently made a hit in authorship. The fact is, until the publication of the present treatise, there was a great hiatus in English literature in regard to the diseases of the rectum and anus, and the flattering reception of this work need therefore not surprise us. The monographs of Mayo and of Syme were able and scientific productions, reflecting great credit upon their authors; but it may truly be affirmed that they were, even at the date of their publication, sadly in arrear of the existing state of the science of which they professed to give an account. Nearly all the other works that had from time to time been issued by the British press were the offspring of charlatanism, designed to entrap the public, and to enrich the pockets of the writers. The treatise of the late Dr. Bushe, an eminent Irish surgeon, settled for some time in New York, where he finally died, was, in fact, the only monograph having the slightest pretensions to completeness, upon the subject in question; the work was issued in 1837, accompanied by an atlas of illustrations, and has long been out of print, although eminently deserving of a new edition. In freely availing himself of the labors of Dr. Bushe, some of the American reviewers took Mr. Ashton severely to task, accusing him of having taken undue liberties, a charge from which his acknowledgments in the preface should have fully screened him.

The present edition of Mr. Ashton's work has been carefully revised, and, to render it still more useful, it has been illustrated by a number of engravings, admirably executed by Mr. Bagg, from original drawings by Mr. Tuson, from cases occurring in the author's practice. The arrangement of the topics is the same as in the previous issues, and no changes have been introduced in regard to the principles of treatment, their soundness and correctness having been confirmed by additional experience.

It would be idle to attempt an analysis of a work that has, in so short a time, reached its third edition. As it now presents itself, we have no hesitation in saying that it is the ablest treatise on the subject in any language. The book, which should be in the library of every practitioner, has recently been reproduced in this city by the eminent publishing house of Messrs. Blanchard & Lea, in a style worthy of the original, both as it respects the paper, the typography, and the illustrations.

